# Chronic kidney disease in high-risk populations: the urgent need to close the diagnostic gap through systematic screening

**DOI:** 10.1590/2175-8239-JBN-2026-E008en

**Published:** 2026-06-22

**Authors:** Joao Roberto de Sa

**Affiliations:** 1 Centro Universitário Faculdade de Medicina do ABC, Disciplina de Endocrinologia e Doenças Metabólicas, Santo André, SP, Brazil.; 2 Universidade Federal de São Paulo, Escola Paulista de Medicina, Centro de Diabetes, Ambulatório de Doença Renal e Transplante, São Paulo, SP, Brazil.

The SEARCH study by Proença de Moraes et al., published in the *Brazilian Journal of Nephrology* ^1^, delivers a com­pelling yet sobering message: despite substantial advances in the understanding and treatment of chronic kidney disease (CKD), early detection in high-risk populations remains markedly inadequate^2,3^.

In this large-scale national investigation involving more than 14,000 adults with diabetes and/or hypertension in Southern Brazil, without prior awareness of CKD, nearly one in five individuals met KDIGO criteria for CKD^4^. This finding transcends epidemiology; it reflects a systemic failure in healthcare delivery. It exposes a striking paradox in the Brazilian healthcare system, where universal access to labo­ratory testing coexists with significant underdiagnosis and poor risk stratification.

Perhaps the most concerning finding of the SEARCH study is not the high prevalence of CKD, but rather the persistently low utilization of albuminuria testing^2,3^. Prior to screening, fewer than 5% of participants had undergone urinary albumin-to-creatinine ratio (UACR) assessment. Even after the identification of abnormal results, testing rates remained below 10%. This represents a critical missed opportunity, particularly in the current therapeutic landscape, in which GLP-1 receptor agonists^5^, sodium-glucose cotransporter-2 inhibitors (SGLT2i)^6,7,8^, and finerenone^9^ have demonstrated consistent renoprotective benefits. Failure to assess albuminuria not only delays diagnosis but also limits the timely initiation of disease-modifying therapies, with substantial long-term consequences for both patients and healthcare systems.

Notably, SEARCH provides a signal—albeit observational—suggesting a potential benefit from early detection. Participants identified with reduced estimated glomerular filtration rate (eGFR) at screening exhibited a slower decline in renal function over the subsequent two years compared with the preceding period. Although causality cannot be inferred, this temporal pattern raises an important hypothesis: early recognition, coupled with appropriate therapeutic intervention, may alter disease trajectories. This is consistent with prior evidence demonstrating that CKD awareness improves adherence to guideline-directed therapy through better documentation, patient engagement, and treatment optimization.

The implications extend beyond individual patient care to the broader public health perspective. Using validated risk prediction models, the authors estimate that approximately 169,000 cases of kidney failure could occur within two years among high-risk individuals in Brazil, increasing to nearly 500,000 within five years^10^. These projections are particularly alarming given that the Brazilian dialysis population has doubled over the past 15 years, underscoring the urgent need for structured and proactive interventions aimed at early disease detection and management.

SEARCH also reinforces the role of established risk factors—including older age, diabetes, obesity, and male sex—associated with reduced eGFR and albuminuria. Interestingly, hypertension demon­strated a stronger association with reduced eGFR than with albuminuria, highlighting the heterogeneity of renal phenotypes in cardiometabolic disease. This finding further supports the use of complementary markers—eGFR and UACR—as recommended by KDIGO 2024 to avoid underestimation of CKD risk^11^.

Several limitations should be acknowledged. Measurements of serum creatinine and UACR were based on single assessments without confirmatory testing, and the study population was predominantly derived from a region with relatively better healthcare access. Nonetheless, these limitations do not detract from the study’s central message: screening for CKD in high-risk populations is feasible, necessary, and potentially effective in modifying disease progression but remains critically underutilized.

The evidence is unequivocal. Without closing the diagnostic gap, advances in nephroprotective therapies will fail to reach those most in need. Measurement of serum creatinine alone is insufficient. Albuminuria testing must evolve from an optional investigation to a mandatory standard of care. Screening strategies should be systematically integrated into chronic disease management^12^, rather than implemented as sporadic awareness initiatives.

The SEARCH study issues a clear call to action for clinicians, policymakers, and healthcare systems. Diagnostic tools are readily available. Effective therapies exist. Clinical guidelines are well established. The remaining challenge lies in implementation, particularly within primary and secondary care settings, where the majority of high-risk individuals are managed. When one in five high-risk patients harbors undiagnosed CKD, the question is no longer whether screening is necessary, but rather how to ensure its consistent and systematic execution.

## Data Availability

No new data were generated or analyzed in this study.
