# Supplementary material for: Chronic kidney disease in high-risk populations: the urgent need to close the diagnostic gap through systematic screening
Source: J Bras Nefrol. 2026 Jun 22;48(2):e2026E008. doi: 10.1590/2175-8239-JBN-2026-E008en (PMC13286654; doi:10.1590/2175-8239-JBN-2026-E008en)
Supplement: Translation

# Translation: Doença renal crônica em populações de alto risco: uma necessidade urgente de reduzir a lacuna diagnóstica por meio do rastreamento sistemático

**DOI:** 10.1590/2175-8239-JBN-2026-E008pt

**Authors:** Joao Roberto de Sa

**Affiliations:** 1 Centro Universitário Faculdade de Medicina do ABC, Disciplina de Endocrinologia e Doenças Metabólicas, Santo André, SP, Brasil.; 2 Universidade Federal de São Paulo, Escola Paulista de Medicina, Centro de Diabetes, Ambulatório de Doença Renal e Transplante, São Paulo, SP, Brasil.

O estudo SEARCH, conduzido por Proença de Moraes et al. e publicado no *Brazilian Journal of Nephrology* ^1^, transmite uma mensagem contundente e, ao mesmo tempo, preocupante: apesar dos avanços substanciais na compreensão e no tratamento da doença renal crônica (DRC), a detecção precoce em populações de alto risco permanece marcadamente inadequada^2,3^.

Neste amplo estudo nacional, envolvendo mais de 14.000 adultos com diabetes e/ou hipertensão no Sul do Brasil, sem conhecimento prévio de DRC, quase um em cada cinco indivíduos preenchia critérios da KDIGO para DRC^4^. Esse achado transcende a epidemiologia; é um reflexo de uma falha sistêmica na prestação de cuidados em saúde. Expõe um paradoxo marcante no sistema de saúde brasileiro, no qual a disponibilidade universal de exames laboratoriais coexiste com um subdiagnóstico significativo e uma estratificação de risco inadequada.

Talvez o achado mais preocupante do estudo SEARCH não seja a elevada prevalência de DRC, mas sim a persistente baixa utilização da avaliação de albuminúria^2,3^. Antes do rastreamento, menos de 5% dos participantes haviam realizado a dosagem da relação albumina/creatinina urinária (UACR). Mesmo após a identificação de resultados alterados, as taxas de testagem permaneceram abaixo de 10%. Isso representa uma oportunidade crítica perdida, especialmente no contexto terapêutico atual, no qual agonistas do receptor de GLP-1^5^, inibidores do cotransportador sódio-glicose tipo 2 (iSGLT2)^6,7,8^ e finerenona^9^ demonstraram benefícios renoprotetores consistentes. A não reali­zação da avaliação de albuminúria não apenas atrasa o diagnóstico, como também limita a introdução oportuna de terapias modificadoras da doença, com consequências substanciais a longo prazo para pacientes e sistemas de saúde.

De forma relevante, o SEARCH oferece um sinal—embora observacional—de possível benefício da detecção precoce. Participantes identificados com redução da taxa de filtração glomerular estimada (TFGe) no momento do rastreamento apresentaram declínio mais lento da função renal nos dois anos subsequentes, em comparação com o período anterior. Embora não seja possível estabelecer causalidade, esse padrão temporal levanta uma hipótese importante: o reconhecimento precoce, associado a intervenções terapêuticas adequadas, pode modificar a trajetória da doença. Esse achado é consistente com evidências prévias de que o reconhecimento da DRC melhora a adesão a terapias baseadas em diretrizes, por meio de melhor documentação clínica, maior engajamento do paciente e otimização do tratamento.

As implicações extrapolam o cuidado individual e alcançam a saúde pública. Utilizando modelos validados de predição de risco, os autores estimam que aproximadamente 169.000 casos de falência renal poderão ocorrer em dois anos entre indivíduos de alto risco no Brasil, número que pode se aproximar de 500.000 em cinco anos^10^. Essas projeções são particularmente alarmantes, considerando que a população em diálise no Brasil dobrou nos últimos 15 anos, o que ressalta a necessidade urgente de intervenções estruturadas e proativas voltadas à detecção e ao manejo precoce da doença.

O estudo SEARCH também reforça o papel de fatores de risco estabelecidos—incluindo idade avançada, diabetes, obesidade e sexo masculino—associados à redução da TFGe e à presença de albuminúria. Notavelmente, a hipertensão mostrou associação mais forte com redução da TFGe do que com albuminúria, evidenciando a heterogeneidade dos fenótipos renais nas doenças cardiometabólicas. Esse achado reforça a importância do uso combinado de marcadores—TFGe e UACR—conforme recomendado pela KDIGO 2024, a fim de evitar a subestimação do risco de DRC^11^.

Algumas limitações devem ser reconhecidas. As dosagens de creatinina sérica e UACR foram realizadas em momento único, sem confirmação sistemática, e a população estudada foi pre­dominantemente oriunda de uma região com melhor acesso a serviços de saúde. Ainda assim, essas limitações não comprometem a principal mensagem do estudo: o rastreamento da DRC em populações de alto risco é factível, necessário e potencialmente eficaz para modificar a progressão da doença; porém, permanece criticamente subutilizado.

A evidência é inequívoca. Sem o fechamento da lacuna diagnóstica, os avanços nas terapias nefroprotetoras não alcançarão aqueles que mais necessitam delas. A dosagem isolada de creatinina é insuficiente. A avaliação da albuminúria deve deixar de ser opcional para tornar-se um componente obrigatório do cuidado de qualidade. Estratégias de rastreamento precisam ser incorporadas de forma sistemática ao manejo das doenças crônicas^12^, em vez de serem implementadas apenas como iniciativas pontuais de conscientização.

O estudo SEARCH constitui um claro chamado à ação para clínicos, gestores e sistemas de saúde. As ferramentas diagnósticas estão disponíveis. As terapias eficazes existem. As diretrizes clínicas são bem estabelecidas. O desafio remanescente reside na implementação—particularmente na atenção primária e secundária, onde a maioria dos pacientes de alto risco é acompanhada. Quando um em cada cinco indivíduos de alto risco apresenta DRC não diagnosticada no momento do rastreamento, a questão já não é se o rastreamento é necessário, mas sim como implementá-lo de forma sistemática e consistente nos cenários em que esses pacientes são atendidos.

## Data Availability

Nenhum dado novo foi gerado ou analisado neste estudo.
